# 

**Published:** 1800-03

**Authors:** 


### NEW MEDICAL PUBLICATIONS IN FEBRUARY.

Instructions for the Relief and Cure of Ruptures; and Advice to Families who have weak, rickety, or deformed Children; by J. EDY, M. D. 2s. 6d. H. D. Symonds.

A View of the Treatment of Ulcers, more especially those of the scrophulous, phagedænic, and cancerous description. With an Appendix on Baynton's new mode of treating old Ulcers of the Legs; by RICHARD NAYLOR, Surgeon to the Gloucester Infirmary, 3s. 6d. boards. Kearsley.

Elements of Chemistry; comprehending all the most important Facts and Principles in the Works of Fourcroy and Chaptal: with the Addition of the more recent Chemical Discoveries which have been made known in Britain and on the Continent; by ROBERT HERON, 12s. boards. Longman and Rees.

Observations on the Diseases which prevailed on board a Part of his Majesty's Squadron, on the Leeward Island Station, between Nov. 1794 and April 1796. By Leonard Gillespie, M. D. Surgeon and Agent to the Naval Hospital at Fort Royal, Martinique, 5s. boards. Cuthell.

The Lady and Gentleman's Botanical Pocket Book; by WM. MAVOR, LL. D. Illustrated with Plates, 4s. 6d. bound. Newbery.

Transactions of the Linnean Society; vol. 5. 1l. 1s. bds. White.

---

### NEW PUBLICATIONS IN FRANCE.

*Nouveau Systême de l'Universe, &c.*—New System of the Universe; or a Philosophical Abridgement of Natural Philosophy and Chemistry; to which are added some new Disoveries of the author, a View of the Affinity of those Sciences to others, and their Application to the Arts in general. By Citizen LEOPOLD MATHIEW, of Nancy, Professor of Natural History and Chemistry at Paris, &c. One Volume, 8vo. price 3 francs, 50 centimes.

The German Medico-Chirurgical Library; being a translation of the best German authors who have written on the Curative Art. Volumes I. and II. contain the Treatise on Herniæ, by G. A. Richter, translated by Rougemont, in two large octavo Volumes, containg 848 pages, price 10 francs. A small number of proof impressions have been worked off on fine wove paper, price 13 francs.

*Abregé d'Anatomie, &c.*—An Abridgment of Anatomy, for the Use of Students in Medicine and Surgery, 2 vol. 12mo. containing 974 pages. Paris, Theophile, and Maquignon, senior.

*Encyclopedie Methodique, &c.*—The Chirurgical Part of the Methodical Encyclopædia. By M. de la Roche, Physician to the regiment of Swiss Guards, Member of the College of Medicine at Geneva, and of the Royal Society of Medicine at Edinburg; and M. Petit Radel, Member of the Faculty of Paris. This part completes the first Volume in 4to. Paris, Panckoucke, rue des Poitevins.

*Observation sur l'Amputation de la Cuisse, &c.*—Observation on the Amputation of the Thigh; necessarily adopted from the spina ventosa of the tibia and the peroneum; and which operation was attended with the greatest success. By Cyprian Bertrand Lagresie, Doctor of Medicine of the Faculty of Montpellier, &c. and Surgeon in Chief to the Armies of the Republic; a pamphlet of 26 pages, with a plate, price 75 centimes. Paris, Migneret, rue Jacob.

*Ouverture du Cadavre, &c.*—Appearances on opening the Body of a young Woman 32 years of age, who died in consequence of an Anasarca, at the beginning of the pains of labour. By Ant. Plachon, Ancient Member of the College of Surgery at Paris; a pamphlet of 16 pages. The author observes, that the antero-posterior diameter of the womb was only 6 centimetres and a half (about 2 inches, 6 lines); the circumference of the womb was 21 centimetres, 6 millimetres (8 inches); and the circumference of the child's head was about 3 decimetres, 2 centrimetres, (12 inches.)

### To CORRESPONDENTS.

Besides the Communications which appear in this Number, we have received those of Drs. Sherwen, Drake, Vage, and Moffman, Messrs. Penkivel, Shorter, White, Brown, A Navy Surgeon, and W. D. D. which last, being in answer to a paper published in the Journal with the Author's name, cannot be admitted without signature.

We have also received, some time ago, an extensive Analysis of the first section of Mr. Brown's Observations on Zoonomia, but have delayed its publication, apprehending it might lead to a controversy on theoretical opinions, inconsistent with our plan.

We are not yet satisfied with any construction of Medical Themometers we have seen; but have no doubt that we shall be able in our next to give a satisfactory answer to those correspondents who wish to be furnished with such instruments.

